# Genetic Aberration Analysis in Thai Colorectal Adenoma and Early-Stage Adenocarcinoma Patients by Whole-Exome Sequencing

**DOI:** 10.3390/cancers11070977

**Published:** 2019-07-12

**Authors:** Thoranin Intarajak, Wandee Udomchaiprasertkul, Chakrit Bunyoo, Jutamas Yimnoon, Kamonwan Soonklang, Kriangpol Wiriyaukaradecha, Wisut Lamlertthon, Thaniya Sricharunrat, Worawit Chaiwiriyawong, Bunchorn Siriphongpreeda, Sawannee Sutheeworapong, Kanthida Kusonmano, Weerayuth Kittichotirat, Chinae Thammarongtham, Piroon Jenjaroenpun, Thidathip Wongsurawat, Intawat Nookaew, Chirayu Auewarakul, Supapon Cheevadhanarak

**Affiliations:** 1 Bioinformatics and Systems Biology Program, School of Bioresources and Technology and School of Information Technology, King Mongkut’s University of Technology Thonburi, Bangkok 10150, Thailand; 2 Bioinformatics Unit for Genomic Analysis, Division of Research and International Relations, HRH Princess Chulabhorn College of Medical Science, Chulabhorn Royal Academy, Bangkok 10210, Thailand; 3 Systems Biology and Bioinformatics Research Group, Pilot Plant Development and Training Institute, King Mongkut’s University of Technology Thonburi, Bangkok 10150, Thailand; 4 Molecular Biology and Genomic Laboratory, Division of Research and International Relations, HRH Princess Chulabhorn College of Medical Science, Chulabhorn Royal Academy, Bangkok 10210, Thailand; 5 Cytogenetics Unit, Central Research Laboratory, Division of Research and International Relations, HRH Princess Chulabhorn College of Medical Science, Chulabhorn Royal Academy, Bangkok 10210, Thailand; 6 Data Management Unit, HRH Princess Chulabhorn College of Medical Science, Chulabhorn Royal Academy, Bangkok 10210, Thailand; 7 Faculty of Medicine and Public Health, HRH Princess Chulabhorn College of Medical Science, Chulabhorn Royal Academy, Bangkok 10210, Thailand; 8 Pathology Laboratory Unit, Chulabhorn Hospital, HRH Princess Chulabhorn College of Medical Science, Chulabhorn Royal Academy, Bangkok 10210, Thailand; 9 Department of Medical Oncology, Chulabhorn Hospital, HRH Princess Chulabhorn College of Medical Science, Chulabhorn Royal Academy, Bangkok 10210, Thailand; 10 School of Bioresources and Technology, King Mongkut’s University of Technology Thonburi, Bangkok 10150, Thailand; 11 Biochemical Engineering and Systems Biology research group, National Center for Genetic Engineering and Biotechnology (BIOTEC) at King Mongkut’s University of Technology Thonburi, Bangkhuntien, Bangkok 10150, Thailand; 12 Department of Biomedical Informatics, College of Medicine, University of Arkansas for Medical Sciences, Little Rock, AR 72205, USA; 13 Department of Biology and Biological Engineering, Chalmers University of Technology, SE-412 96 Gothenburg, Sweden; 14 Department of Physiology and Biophysics, College of Medicine, The University of Arkansas for Medical Sciences, Little Rock, AR 72205, USA

**Keywords:** somatic mutation, colorectal adenoma, early-stage colorectal adenocarcinoma, screen marker

## Abstract

Colorectal adenomas are precursor lesions of colorectal adenocarcinoma. The transition from adenoma to carcinoma in patients with colorectal cancer (CRC) has been associated with an accumulation of genetic aberrations. However, criteria that can screen adenoma progression to adenocarcinoma are still lacking. This present study is the first attempt to identify genetic aberrations, such as the somatic mutations, copy number variations (CNVs), and high-frequency mutated genes, found in Thai patients. In this study, we identified the genomic abnormality of two sample groups. In the first group, five cases matched normal-colorectal adenoma-colorectal adenocarcinoma. In the second group, six cases matched normal-colorectal adenomas. For both groups, whole-exome sequencing was performed. We compared the genetic aberration of the two sample groups. In both normal tissues compared with colorectal adenoma and colorectal adenocarcinoma analyses, somatic mutations were observed in the tumor suppressor gene *APC* (Adenomatous polyposis coli) in eight out of ten patients. In the group of normal tissue comparison with colorectal adenoma tissue, somatic mutations were also detected in Catenin Beta 1 (*CTNNB1*), Family With Sequence Similarity 123B (*FAM123B*), F-Box And WD Repeat Domain Containing 7 (*FBXW7*), Sex-Determining Region Y-Box 9 (*SOX9*), Low-Density Lipoprotein Receptor-Related Protein 5 (*LRP5*), Frizzled Class Receptor 10 (*FZD10*), and AT-Rich Interaction Domain 1A (*ARID1A*) genes, which are involved in the Wingless-related integration site (Wnt) signaling pathway. In the normal tissue comparison with colorectal adenocarcinoma tissue, Kirsten retrovirus-associated DNA sequences (*KRAS*), Tumor Protein 53 (*TP53*), and Ataxia-Telangiectasia Mutated (*ATM*) genes are found in the receptor tyrosine kinase-RAS (RTK–RAS) signaling pathway and p53 signaling pathway, respectively. These results suggest that *APC* and *TP53* may act as a potential screening marker for colorectal adenoma and early-stage CRC. This preliminary study may help identify patients with adenoma and early-stage CRC and may aid in establishing prevention and surveillance strategies to reduce the incidence of CRC.

## 1. Introduction

Colorectal cancer (CRC) is the third most common cancer worldwide, with increasing numbers of estimated new cases in both males and females [1]. Approximately 0.45% of the population in the USA is diagnosed with CRC [2,3], which is the third most common cancer in the USA. CRC is the second most incident cancer in Thailand. The five-year survival rates of CRC in the early-stage and advanced-stage in males and females are approximately 63%–92% and 11%–89%, respectively [4]. It has been reported that the majority of diagnosed CRC patients in Thailand have advanced-stage cancer (70.80%), with an overall survival rate of 5% [4,5]. Previous studies reported that new CRC cases in Thailand increased by 8.68% and 6.86% in males and females, respectively [5,6]. These results indicated that an effective screening program is necessary for the prevention of CRC in the Thai population [5,7,8]. Therefore, accumulation for mutation information related to CRC in the Thai population by screening for colorectal adenoma, which is the precursor lesion for CRC, and diagnosis of early-stage CRC are both very important for CRC prevention [9,10].

The development of CRC is a complex and heterogeneous process. The transformation of normal colon tissue to a CRC sequence is known to be caused by several genetic aberrations, such as mutations that inactivate tumor suppressor gene function, chromosomal instability, and DNA methylation alteration [11,12,13,14]. The progression from normal tissue to colorectal cancer can be classified into two pathways, which are the traditional pathway and alternative pathway. As for the traditional pathway, the normal cell developed tubular adenomas followed by the development of colorectal cancer through the Wnt signaling pathway, mitogen-activated protein kinases (MAPK) pathway, phosphoinositide 3-kinase (PI3K) signaling pathway, transforming growth factor-β (TGFβ) signaling pathway, and p53 signaling pathway. The alternative pathway involves sessile serrated polyps and their progression to colorectal cancer by the same sequential pathway as the traditional pathway [11]. Recent advances in DNA sequencing technology have enabled a better understanding of the molecular basis of CRC pathogenesis [13,15,16,17,18]. Comparison of the genetic profile between normal, colorectal adenoma, and CRC in Chinese CRC patients by exome capture sequencing identified somatic gene mutations involved in the Wnt signaling pathway, cell adhesion, and ubiquitin-mediated proteolysis pathway [19]. In examining the progression of normal cells to colorectal adenoma and adenocarcinoma and searching for potential molecular markers, studies have identified differences in driver mutations in sessile serrated adenoma compared with conventional adenoma [20,21]. However, results from a study of African American CRC patients showed different patterns of somatic gene mutations compared with mutations derived from The Cancer Genome Atlas (TCGA) data [22].

A recent attempt to establish a screening protocol in the Thai population during July 2009–June 2010 examined new potential CRC screening methods in 1404 healthy volunteers using a fecal occult blood test (FOBT) and fecal immunochemical tests (FITs). The obtained results were compared with the screening results by colonoscopy. The study suggested the integration of colonoscopy into the national screening approach for the detection of early-stage CRC [8]. Although the gold standard of CRC early detection is a colonoscopy, this strategy is not cost-effective at the population screening level [23] and may be prone to causing infection by pathogenic bacteria and virus, such as hepatitis B and C, prion disease, *Salmonella* spp. and HIV [24]. A non-invasive approach can eliminate the risk of infectious disease during the examination. However, methods such as whole-genome sequencing are still expensive. Thus, a non-invasive screening technique with high sensitivity and specificity for early-stage colorectal adenoma and CRC is required [25,26,27].

In general, the treatment of colorectal cancer is chemotherapy, radiotherapy, adjuvant therapy, and surgery [11]. The response rate of the first-line drug which was used in the metastasis stage is 20% [28]. Moreover, the second-line drug which targeted the RAS wild-type has a higher response rate but the side effects may be harmful to patients and affect their quality of life [28]. Recent studies revealed the mechanism of the new therapeutic targets that are involved in cell proliferation, tumor progression, apoptosis, drug resistance, and autophagy [29,30,31,32,33,34,35]. Inhibition of TGF-β1, FAHFA, which protects tumors from apoptosis, resulting in an enhanced CRC treatment response [31,34]. In addition, the reduction of drug resistance, proliferation, and cancer progression due to silence expression and function of MAGL, HuR, CDC6, and TPC1 represent an innovative therapeutic approach [29,30,32,35]. Moreover, the five-year survival rate of early stage of colorectal cancer is 90% but reduces to 14%–71% in advanced stage [36]. It was found that only 39% of CRC patients were detected in early-stage CRC. This information indicated that increased efficiency of early screening may possibly increase the five-year survival rate and decrease the mortality rate. Therefore, a precise early detection process is vital.

The aim of this study was to identify the genetic abnormalities in genes associated with a high susceptibility of CRC from matched normal, colorectal adenoma, and CRC samples of Thai CRC patients using exome sequencing analysis for potential application as a noninvasive screening marker, such as the amplification of specific genes from stool DNA followed by mutation detection for early screening of precancerous and early-stage CRC.

## 2. Results

### 2.1. Identification of Gene with Somatic Mutations in Normal-Colorectal Adenoma and Normal-CRC in CRC Patients

**Figure 1:**
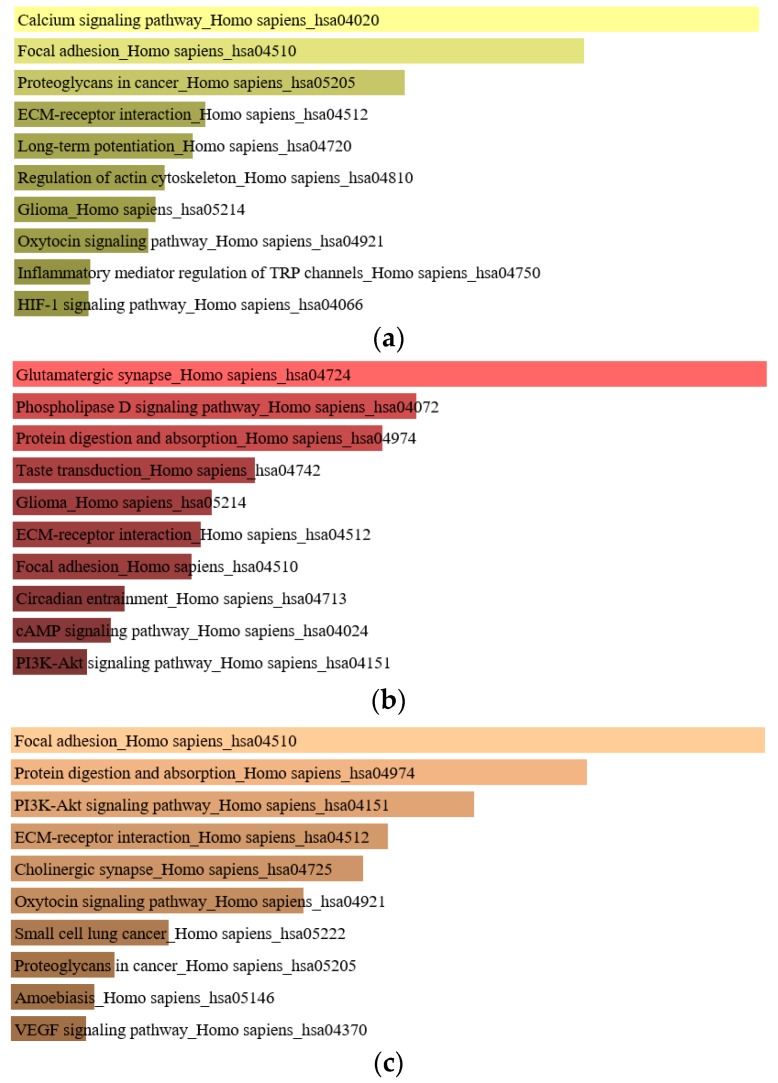
Enriched genes of (**a**) normal-colorectal adenoma, (**b**) normal colorectal cancer (CRC), and (**c**) the intersection of normal-colorectal adenoma and normal-CRC.

Genes with somatic mutations are mutations that occur in cancer cells and not healthy cells [37], and represent one of the main factors that lead to cancer [38]. A total of 2044 genes with somatic mutations were identified in matched normal-colorectal adenoma, which show enrichment in the calcium signaling pathway, focal adhesion pathway, and proteoglycan in the cancer pathway. A total of 1045 genes with the somatic mutation were classified in matched normal-CRC with enrichment in the glutamatergic synapse pathway, phospholipase D signaling pathway, and protein digestion and absorption pathway. The intersection of a gene with somatic mutations between the matched normal-colorectal adenoma and matched normal-CRC were enriched in the focal adhesion pathway, protein digestion and absorption pathway, and PI3K-Alt signaling pathway (Figure 1).

**Figure 2:**
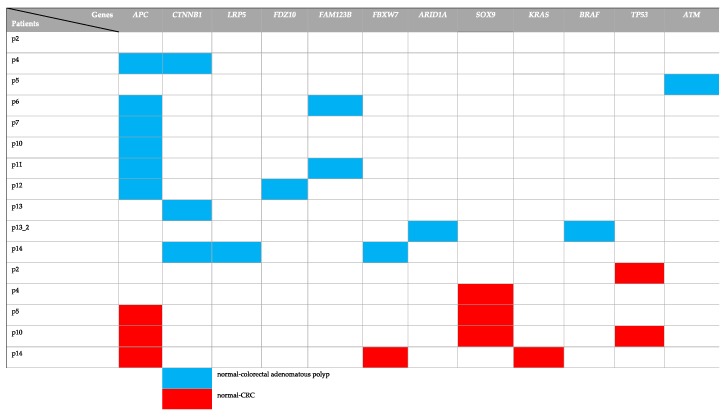
Comparison of the alter gene between The Cancer Genome Atlas (TCGA) and Thai patients. The rows show the list of patients. The columns show the list of gene names.

To determine whether the genes with somatic mutations identified from normal-colorectal adenoma and normal-CRC have been reported in other population. The genes with somatic mutations identified from normal-colorectal adenoma and normal-CRC were compared with those of TCGA, African American, and Chinese CRC data. The result revealed different identified genes in the three groups, except *APC* genes [22]. The African American CRC patient study showed somatic mutations in genes *APC*, *KRAS*, Fc Receptor Like 5 (*FCRL5*), obscurin, cytoskeletal calmodulin and titin-interacting RhoGEF (*OBSCN*), Retinitis Pigmentosa 1 Like 1 (*RP1L1*), Dynein Axonemal Heavy Chain 17 (*DNAH17*), Zinc Finger Protein 568 (*ZNF568*), and Calcium Voltage-Gated Channel Subunit Alpha1 C (*CACNA1C*) [22]. Based on the TCGA data, the identification of a somatic alteration in CRC was performed by many omics scales within 276 samples. The TCGA analysis result identified gene mutations in *APC*, *TP53*, *SOX9*, *KRAS*, *PIK3CA*, *TTN*, *FBXW7*, and *SMAD4* [13]. The genes with the somatic mutation were compared with TCGA data [13,22] (Figure 2). We found that the identified genes with the somatic mutation from TCGA data showed a minor overlap with those identified from normal-colorectal adenoma and normal-CRC samples. The Chinese study performed whole-exome sequencing for both matched normal-colorectal adenoma and matched normal-CRC to identify the genes with somatic mutations. The matched normal-colorectal adenoma result showed the genes with somatic mutations in *APC*, *AMA3*, *OR6X1*, *NMBR*, *EFR3A*, *RBFOX1*, *CDH20*, *BIRC6*, *KRT84*, *SLC15A3*, *FTHL17*, and *GLCCI1*. The matched normal-CRC revealed the genes with somatic mutation in *APC, FBXW7, FLT4, GSK3A, ZFP64, NRXN3, TGM7, GRIK1, KIF25, DTL, GNAL, ATF2, OR51E2, CUX1, PPAP2C, CORO1A, OR13J1, KRTAP19-7, POU4F3, PPP1R3C, NARS2, NFATC2, FAM109A, FAM54A, TFR2, ZNF781, RRP8, ZFP36L2, KRT31, RYR1, KIAA1409, NRG1, PGM1, ALPK1, FAM181A, FCRL3*, and *SDK1* [19]. Colorectal adenoma samples of p4, p6, p7, p10, p11, p12 exhibit mutations in *APC*, while p4, p13, and p14 show mutations in *CTNNB1.* p6 and p11 show mutations in *FAM123B*. In addition, CRC samples of p5, p10, and p14 reveal the mutation in *APC*. As for CRC samples of p4, p5, and p10, the mutations of the *SOX9* gene were found. While CRC of p2 and p10 exhibit mutations in the *TP53* gene (Table S2). Finally, mutations in the *FBXW7* and *KRAS* genes are identified in CRC of p14 (Figure 2).

**Figure 3:**
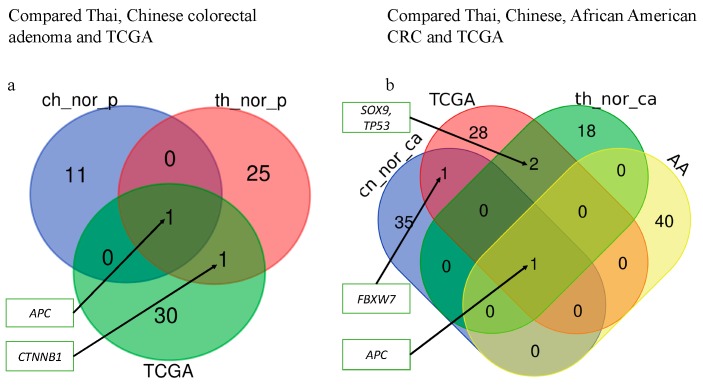
Comparison of high-frequency mutated genes. (**a**) The comparison of genes with somatic mutations from matched normal-colorectal adenoma in Thai CRC samples (th_nor_p), matched normal-colorectal adenoma in Chinese CRC samples (ch_nor_p) and matched normal-CRC in the TCGA sample; (**b**) the comparison of genes with somatic mutations in matched normal-CRC of Thai people (th_nor_ca), matched normal-CRC in Chinese people (ch_nor_ca), TCGA, and African American (AA) CRC sample.

In addition, comparison of the genes with somatic mutations in the normal-CRC, normal-colorectal adenoma groups, the Chinese study, the TCGA data, and African American CRC patients demonstrated that *SOX9* and *TP53* genes were common genes in the TCGA and Thai normal-CRC data, whereas the *CTNNB1* gene was a common gene in the Thai normal-colorectal adenoma and TCGA data. Moreover, the *FBXW7* gene was a common gene in Chinese normal-CRC and TCGA (Figure 3) (Table 1). Interestingly, *APC* was found as a common gene in the four groups.

### 2.2. Copy Number Variation (CNVs)

**Figure 4:**
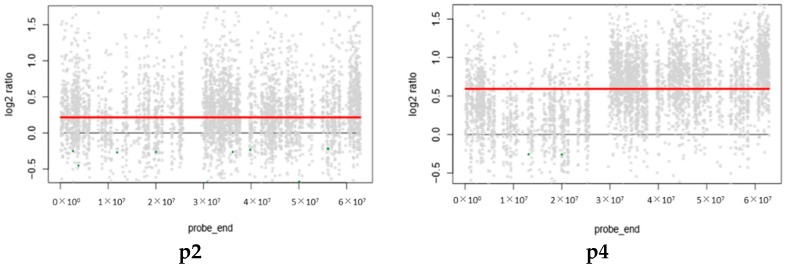

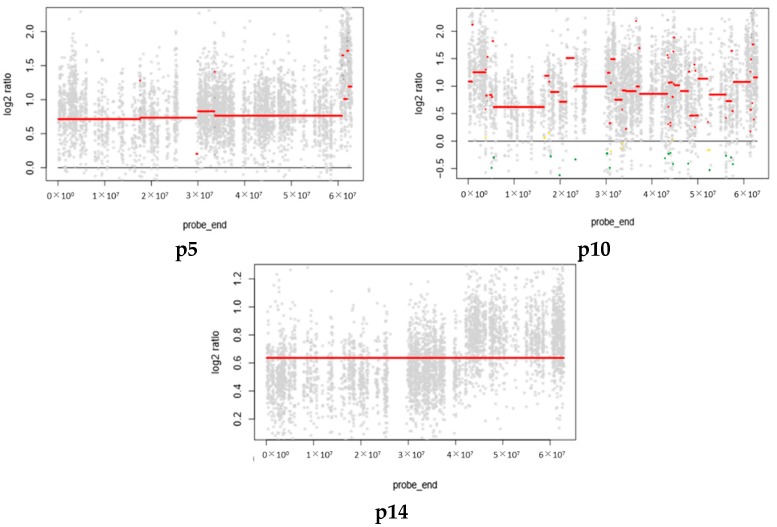
The gain of copy number variation (CNV) at chromosome 20. Patients p4, p5, p10, and p14 showed the gain of chromosome 20, beside patient p2, who did not pass the criteria.

CNVs are structural variations due to chromosome alterations, including duplication or deletion of regions in the genome, that lead to carcinogenesis in tumor patients [39,40]. Early-stage CRC can be detected by the gain of chromosomes 8q, 13, and 20q and loss of chromosomes 8p, 17p, and 18q [41]. Other studies have identified CNVs using plasma and CRC tissues. The finding exhibited the CNVs gain chromosome 20, position 20q12, and the CNVs loss in chromosome 8, position 8p23.1 to 8p23.2 [42]. Therefore, the identification of CNVs may yield more potential markers for further investigation. Here, we identified CNVs from matched normal-colorectal adenoma and normal-CRC only in the autosome. The results revealed the gain of chromosome 20 in four patients (Figure 4). These findings suggest that the gain of chromosome 20 in, for example, the *BCL2L1*, *TPX2*, *SRC*, *AURKA*, and *GNAS* genes [43] may be a potential marker for the detection of early-stage CRC (Table S1).

### 2.3. The Analysis of Enriched Genes in Normal-Colorectal Adenoma and Normal-CRC Groups

Since colorectal cancer development is involved with several signaling pathways. The analysis of enriched genes provides more information and a better understanding of the colorectal cancer development process. The identified somatic mutation genes in normal-colorectal adenoma are enriched in the calcium signaling pathway, focal adhesion, protein digestion, proteoglycans in cancer, and the extracellular matrix (ECM)-receptor pathway. The somatic mutation genes in normal-CRC are enriched in the glutamatergic synapse, phospholipase D signaling pathway, protein digestion and absorption, taste transduction, glioma, ECM-receptor interaction and focal adhesion pathway (Figure 1). The focal adhesion and ECM-receptor pathways play a key role in cancer progression, migration, proliferation, survival, and apoptosis of tumor cells [44,45].

### 2.4. Candidate Genes with Driver Mutation in Normal-Colorectal Adenoma and Normal-CRC Groups

**Figure 5:**
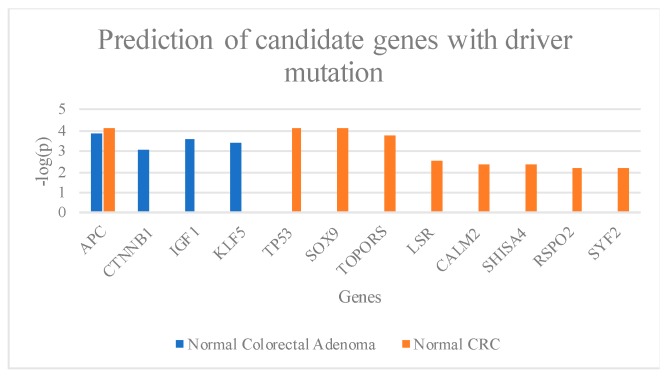
Prediction of candidate genes with driver mutations from normal-colorectal adenomatous polyps and normal-CRC.

Candidate genes with driver mutations are genes that participate in the abnormality of cell growth in cancer cells but are not involved in carcinogenesis [38]. It appears that the individual who presents genes with driver mutations may have a greater chance of developing colon cancer. Therefore, the identification of genes with driver mutations in colorectal adenoma and early-stage CRC may help to prevent CRC and improve the survival rate and the quality of life of CRC patients. Our results showed that *APC*, *CTNNB1*, *IGF1*, and *KLF5* (Figure 4) were frequently mutated in normal-colorectal adenoma. The result shows four stop gains and three frameshift variants in the *APC* gene. The CTNNB1 gene contained three missense variants, while *IGF1* and *KLF5* genes are missense variants (Table S3). The normal-CRC showed mutations in *APC*, *TP53*, *SOX9*, *TOPORS*, *LSR*, *CALM2*, *SHISA4*, *RSPO2*, and *SYF2* (Figure 5).

The *APC* genes contain one stop gain, one frameshift variant, and one splice region variant. The two frameshift variants are detected in *TP53* gene. The *SOX9* gene contains one missense variant and two frameshift variants. The *TOPORS* gene shows one stop gain and one frameshift variant. One missense and frameshift variant are found in the *LSR* gene. The *CALM2* gene shows only one splicing region variant. The *SHISA4* gene carries in-frame deletion. The *RSPO2* gene includes two splice region variants. The *SYF2* gene consists of one frameshift variant (Table S3).

## 3. Discussion

In the last thirty years, the global pattern of incidence and mortality trends of CRC have been divided into three groups: (1) increase of both incidence and mortality, (2) increase in incidence but decrease in mortality, and (3) decrease of both incidence and mortality [46]. The reduction of the mortality rate in groups 2 and 3 is better due to better standard treatment and early detection of colorectal adenoma and CRC. It is suggested that improved screening methods may increase the incidence rate, subsequently reducing the mortality rate in the long-term [46,47,48,49,50,51,52,53].

The progression of normal epithelial cells to CRC involves multiple gene mutations within several signaling pathways, such as the Wnt signaling pathway, MAPK signaling pathway, PI3K signaling pathway, TGFβ signaling pathway, and p53 signaling pathway [11]. While *APC, CTNNB1, LSR, TOPORS, KLF5, IGF1* and *SOX9* are related to Wnt signaling pathways, *KRAS* and *BRAF* are involved in RAS signaling pathways [54,55,56,57,58,59,60]. This indicates that the abnormalities of proliferative pathways may crucial in early colorectal cancer development. Additionally, the defects of tumor suppressor genes such as *ATM* and *TP53* of the P53 signaling pathways are also at the pivot point of the colorectal tumorigenesis [61,62]. In addition, the *CALM2, SHISA4, RSPO2*, and *SYF2* contributed to cell proliferation [63,64,65,66,67]. However, the role of these genes in the cancer development mechanism has not been elucidated.

Here, we identified mutated genes with somatic mutations in adenoma and CRC samples compared with matched normal samples that are involved in important signaling pathways, including the Wnt and p53 signaling pathways. We identified genes with somatic mutations in the normal-colorectal adenoma samples, *APC*, *CTNNB1*, *LRP5*, *FBXW7*, and *ATM* (Figure 2), which overlapped with the TCGA data. Interestingly, the position c.4348C>T (p.R1450*) of the *APC* gene was found in 2 out of 11 patients in the matched normal-colorectal adenoma tissue. The point mutation was reported as the most common mutation of CRC in previous studies from Tunisia and Iran [68,69]. Moreover, this mutation position was identified as the most frequently mutated position in the colorectal adenoma studied from the United Kingdom, Czech Republic, and the Netherlands also [70]. We also identified genes with somatic mutations in normal-CRC samples, *APC*, *FBXW7*, *SOX9*, *KRAS*, and *TP53* (Figure 2), that overlapped with the TCGA data analysis. All of the identified genes in the present study are the members of the Wnt, p53, and RTK–RAS signaling pathways. The exome sequences obtained from the formalin-fixed, paraffin-embedded tissues of the matched normal-colorectal adenoma and normal-CRC revealed 99.85% coverage in the target region. Analysis of high-frequency mutated genes showed 12 known gene mutations in colon cancer-associated pathways, including *APC, TP53, SOX9, TOPORS, IGF1, KLF5, LSR, CALM2, CTNNB1, RSPO2, SYF2*, and *SHISA4*. The normal-colorectal adenoma somatic mutation analysis identified mutations in two key genes, *APC* and *CTNNB1*, which are known to be involved in the Wnt signaling pathway. *IGF1* contributes to the cell cycle progression and inhibits the apoptosis pathway [71]; *IGF1* also promotes cell growth in CRC by activating the *VEGF* gene and, therefore, supports cancer progression of human colon cancer cells [72]. In addition, *KLF5* has been reported as an oncogene that suppresses cancer cell growth and is involved in tumor progression in CRC mouse models [73,74,75,76] (Figure 4). Moreover, a recent study reports that the somatic mutation c.910C>A (p.P304T) position of *KLF5* was reported as the hotspot of mutations in the phosphor–degron domain, which promotes cancer cell proliferation [58].

Based on our finding, the normal-CRC showed the candidate genes, including *APC*, *TP53*, *SOX9*, *TOPORS*, *LSR*, *CALM2*, *SHISA4*, *RSPO2, FBXW7*, and *SYF2*. The *APC*, *SOX9*, *SHISA4*, and *RSPO2* genes are members of the Wnt signaling pathway [11,65,74,77,78] (Figure 5). The *SOX9* gene is overexpressed in both colorectal adenoma and CRC samples compared with normal tissues by 2- and 3.5-fold, respectively [79,80]. *SOX9* is not only the downstream regulator and effector of the Wnt signaling pathway but also increases cell proliferation and transformation [80].

Gene function analysis revealed that genes were predominantly enriched in the ECM-receptor interaction and focal adhesion pathways. Integrins directly interact with the components of ECM and contribute to cell motility and invasion. Studies have demonstrated the crucial role of integrins in regulating tumor cell progression and metastasis by increasing tumor cell migration, invasion, proliferation, and survival. Integrin-mediated migration requires focal adhesion kinase (FAK)-Src family kinase (SFK) signaling [44], which are the main kinases in focal adhesion signaling, followed by recruitment of the proteins necessary for focal adhesion development. Tumor cells display highly altered focal adhesion dynamics that may emulate the development and progression of cancer [45].

Wnt/B-catenin is an important signaling cascade involved in both colorectal development and carcinogenesis [81]. The mutations in this pathway are involved in the first step of progress from normal to colorectal adenoma. [82]. Mutation in the p53 signaling pathway is closely associated with the progression of CRC [11]. *APC* is a tumor suppressor gene and has many functions that affect the progression of cancer cells, such as proliferation, migration, cytoskeletal maintenance, and chromosome instability [83,84]. The *TP53* gene plays an important function in either cell proliferation or trigger senescence and apoptosis [85]. Evidence for the roles of *APC*, *TP53*, and *KRAS* as early driver genes has been demonstrated in several reports [62,86]. Mutated *APC*, *TP53*, and *KRAS* have been identified in colon adenoma as well as in CRC [62,86]. Our results suggest that the gene set identified in the Wnt, p53, and RTK–RAS signaling pathways might be used as a candidate precancerous and early-stage screening marker for CRC. However, it could not be concluded that the genes with driver mutations promote the tumorigenesis because, after surgical resection, none of the patients developed colorectal cancer (the median follow-up period was 6.5 years).

Previous studies showed the gain of chromosome 20 in CRC patients and demonstrated its use as a marker to detect early-stage CRC [41,42]. Our results in Thai CRC patients revealed a gain of chromosome 20 in four out of five samples, in agreement with the previous reports (Figure 4). This suggests that the gain of chromosome 20 in CRC patients could act as a marker for the early detection of CRC.

## 4. Materials and Methods

Here, we identified variants, somatic mutation genes, high-frequency mutated genes, gene list enrichment, and copy number variations (CNVs) following the study flow shown in Figure S1.

### 4.1. Samples

The patients included in this study were in a CRC screening cohort that underwent colonoscopy at Chulabhorn hospital between July 2009 and June 2010. The present study was approved by the Human Research Ethical Committee of the Chulabhorn Research Institute. All formalin-fixed, paraffin-embedded specimens of colorectal adenoma and adenocarcinoma were obtained from the pathology laboratory unit. The specimens were reviewed by the pathologist to confirm the diagnosis and distinguish between low-grade dysplasia and high-grade dysplasia before performing microdissection.

**Figure 6:**
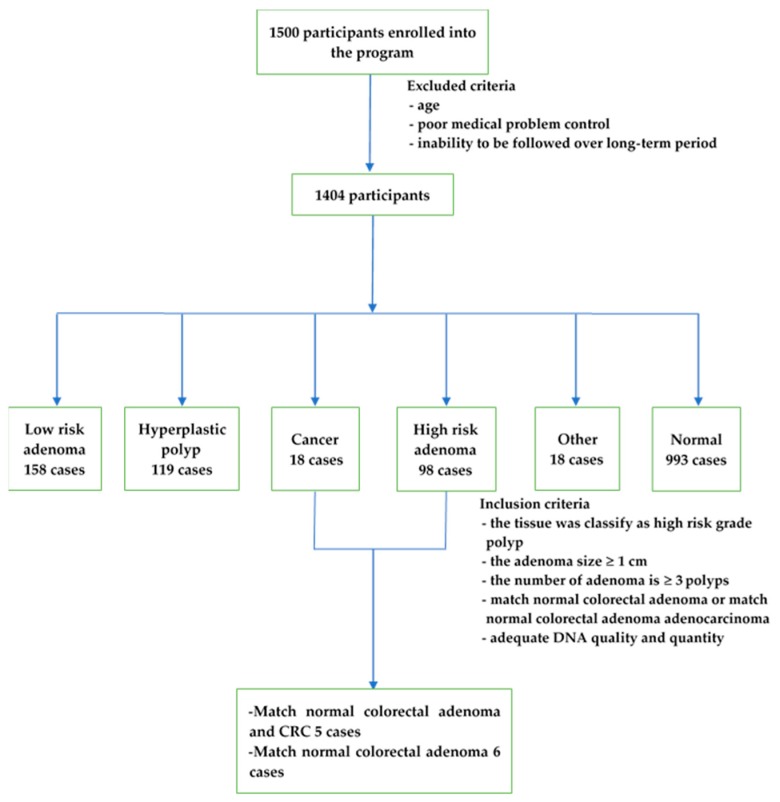
Data flow of participants with the inclusion and exclusion criteria (modified from [8]).

A total of 1500 participants were enrolled in the project (Figure 6). The exclusion criteria included age, poor medical problem control, and the inability to be followed during the study, resulting in 1404 participants [8]. The inclusion criteria were set as (i) colorectal adenoma tissue classified as a high-risk grade, either villous or tubulovillous or sessile serrated polyps, (ii) colorectal adenoma size greater or equal to 1 cm, (iii) the number of colorectal adenomas greater than or equal to 3 polyps, and (iv) matched normal and CRC samples were available. In total, a small cohort of the 5 cases with matched normal and colorectal adenoma tissue and CRC tissue, as well as the 6 cases with matched normal and colorectal adenoma tissue samples, were selected. Patients p2, p4, p6, p7, p10, p11, p12, p13, and p14 had tubular adenoma, while patients p5 and p13_2 had serrated adenoma (Table 2). Among the cases with CRC samples, p4, p5, p10, and p14 showed stage IIA CRC and p2 showed stage I CRC. The locations of most of the specimens were on the left side of the colon, except for the specimen for p10, which was located on the right side.

### 4.2. DNA Extraction and Library Preparation

Genomic DNA was extracted from formalin-fixed, paraffin-embedded tissue using a QIAmp DNA Microkit (Qiagen, Hilden, Germany). The DNA quality and quantity were determined using a NanoDrop ND-1000 (Nanodrop Technology, Wilmington, DE, USA) and Qubit^®^ 2.0 Fluorometer (Life Technologies, Carlsbad, CA, USA), respectively. Exome sequencing was performed by Macrogen (Seoul, Korea) using the SureSelect human all exon kit V4+UTR (Agilent Technologies, Santa Clara, CA, USA), and the exome library of 101 bases paired reads were sequenced using the Illumina HiSeq2000 (Illumina, San Diego, CA, USA) at 100 reads coverage.

### 4.3. Whole-Exome Analysis

To identify point mutations and somatic mutations, the raw FASTQ files were trimmed by trimmomatic [87], then aligned to a human reference genome (GRCh37) by Burrows-Wheeler Alignment Tool (BWA) [88]; duplicate reads were removed by Picard tools [89] and variant calling was performed by the Genome Analysis Toolkit pipeline [90]. Variants were filtered by two criteria: read coverage >50-fold coverage and Phred score >30. The genes with the somatic mutations were predicted in matched normal colorectal adenoma and tumors using MuTect2 [91]. Genes with somatic mutations were filtered by the depth coverage >20-fold coverage. Somatic mutation annotation was performed by Variant Effect Predictor [92]. Default parameters were used in all software analyses.

### 4.4. CNV Analysis

CNV was identified in matched normal-colorectal adenoma and normal-CRC samples using “ExomeCNV” [93] in the R package with default parameters. The criteria to identify gain or loss of copy number variation used absolute log_2_ of ratios >0.5 [94] with default parameters.

### 4.5. The Analysis of Enriched Genes

The analyses of enriched genes in the colorectal adenoma and adenocarcinoma were performed by the use of genes with somatic mutations in the matched normal-colorectal adenoma and matched normal-CRC. Enrichr was used to identify the enriched genes and the enrichment pathway [95,96].

### 4.6. The Genes with Driver Mutation Analysis

The matched normal-colorectal adenoma and matched normal-CRC data were used to identify genes with driver mutations using the MutSigCV application with the default parameters [17].

### 4.7. Availability of Data/Materials

The raw data can be found at the Sequence Read Archive (SRA), accession number PRJNA494574.

## 5. Conclusions

To identify markers for the early detection of CRC, we explored the genetic alterations in six cases with matched normal colorectal adenoma and five cases with matched normal colorectal adenoma CRC. We performed whole-exome sequencing and bioinformatic analysis focused on CNV, somatic mutations, and candidate genes with driver mutations. Our discoveries showed that the gene sets identified in both matched normal colorectal adenoma and matched normal colorectal adenoma and the CRC group are involved in the Wnt, p53, and RTK–RAS signaling pathways. It might be used as a precancerous and early-stage screening marker candidate for CRC.

However, this finding should be validated in a large sample size. One limitation of the current study is its small sample size, and thus more samples in a larger analysis are required for future studies to validate our findings.

## Figures and Tables

**Table 1:** Genes with somatic mutations identified in this study that overlap with TCGA, Chinese, and African American CRC patient data.

Patient	Chr	Start	Stop	Reference	Alternative	Gene	Mutation	Exonic Function	Protein Change	Known
p5t	5	112174631	112174631	C	T	*APC*	SNV	Stop gain	p.Arg1114Ter	rs121913331, COSM13125
p10t	5	112151185	112151185	T	G	*APC*	SNV	Splice Region variant	-	
p14t	5	112155021	112155022	TG	-	*APC*	deletion	Frame shift variant	p.Met431ArgfsTer12	
p4low	5	112175639	112175639	C	T	*APC*	SNV	Stop gain	p.Arg1450Ter	rs121913332, COSM13127
p6low	5	112174249	112174249	T	A	*APC*	SNV	Stop gain	p.Tyr986Ter	
p7low	5	112174129	112174129	A	-	*APC*	deletion	Frame shift variant	p.Cys947ValfsTer8	
p7low	5	112175255	112175255	G	T	*APC*	SNV	Stop gain	p.Glu1322Ter	
p10low	5	112175390	112175391	-	A	*APC*	Insertion	Frame shift variant	p.Thr1368AspfsTer7	
p11low	5	112175639	112175639	C	T	*APC*	SNV	Stop gain	p.Arg1450Ter	rs121913332, COSM13127
p12low	5	112155031	112155032	-	A	*APC*	Insertion	Frame shift variant	p.Asn436LysfsTer8	
p2t	17	7578510	7578510	G	-	*TP53*	Deletion	Frame shift variant	p.C141Afs*29	COSM69019
p10t	17	7576889	7576890	-	T	*TP53*	Insertion	Frame shift variant	p.K320Efs*17	
p4t	17	70119862	70119863	-	TTCGA	*SOX9*	Insertion	Missense mutation	p.V291Sfs*94	
p5t	17	70119855	70119856	-	CGAGA	*SOX9*	Insertion	Frame shift variant	p.F289Rfs*96	
p10t	17	70118889	70118889	A	A	*SOX9*	SNV	Frame shift variant	p.F154Y	
p4low	3	41274898	41274898	G	C	*CTNNB1*	SNV	Missense mutation	p.Trp383Ser	
p13low	3	41266124	41266124	A	G	*CTNNB1*	SNV	Missense mutation	p.Thr41Ala	rs121913412
p14low	3	41266097	41266097	G	A	*CTNNB1*	SNV	Missense mutation	p.Asp32Asn	rs28931588
p14low	4	153258983	153258983	G	A	*FBXW7*	SNV	Stop gain	p.Arg278Ter	
p14t	4	153268102	153268102	C	-	*FBXW7*	Deletion	Frame shift variant	p.Glu236AsnfsTer3	

Chr = Chromosome.

**Table 2:** The clinical information of colorectal adenomatous and CRC patient.

Patient	Gender/Age	Adenoma	Adenocarcinoma	Stage/TNM	LVI	Location
p2	M/65	TA	A	I/pT2N0M0	Y	L
p4	M/55	TA	A	IIA/pT3N0M0	N	L
p5	F/58	SA	A	IIA/pT3N0M0	Y	L
p6	M/64	TA	-	-	N	L
p7	M/58	TA	-	-	N	L
p10	F/56	TA	A	IIA/pT3N0M0	Y	R
p11	M/58	TA	-	-	N	L
p12	M/60	TA	-	-	N	L
p13	M/61	TA	-	-	N	L
p13_2	M/61	SA	-	-	N	L
p14	M/66	TA	A	IIA/pT3N0M0	N	L

M = male, F = female, TA = Tubular adenoma, SA = Serrated adenoma, A = adenocarcinoma, L = left, R = right, LVI = Lymphovascular Invasion, Y = Yes, N = No.

## Supplementary Materials

The following are available online at https://www.mdpi.com/2072-6694/11/7/977/s1, Figure S1: Data flow of the project, Table S1: Cancer related genes in chromosome 20, Table S2: Somatic mutation master table, Table S3: Predicted candidate genes with driver mutation.
