# Supplementary figures and images for: Genetic Aberration Analysis in Thai Colorectal Adenoma and Early-Stage Adenocarcinoma Patients by Whole-Exome Sequencing
Source: Cancers (Basel). 2019 Jul 12;11(7):977. doi: 10.3390/cancers11070977 (PMC6679221; doi:10.3390/cancers11070977)

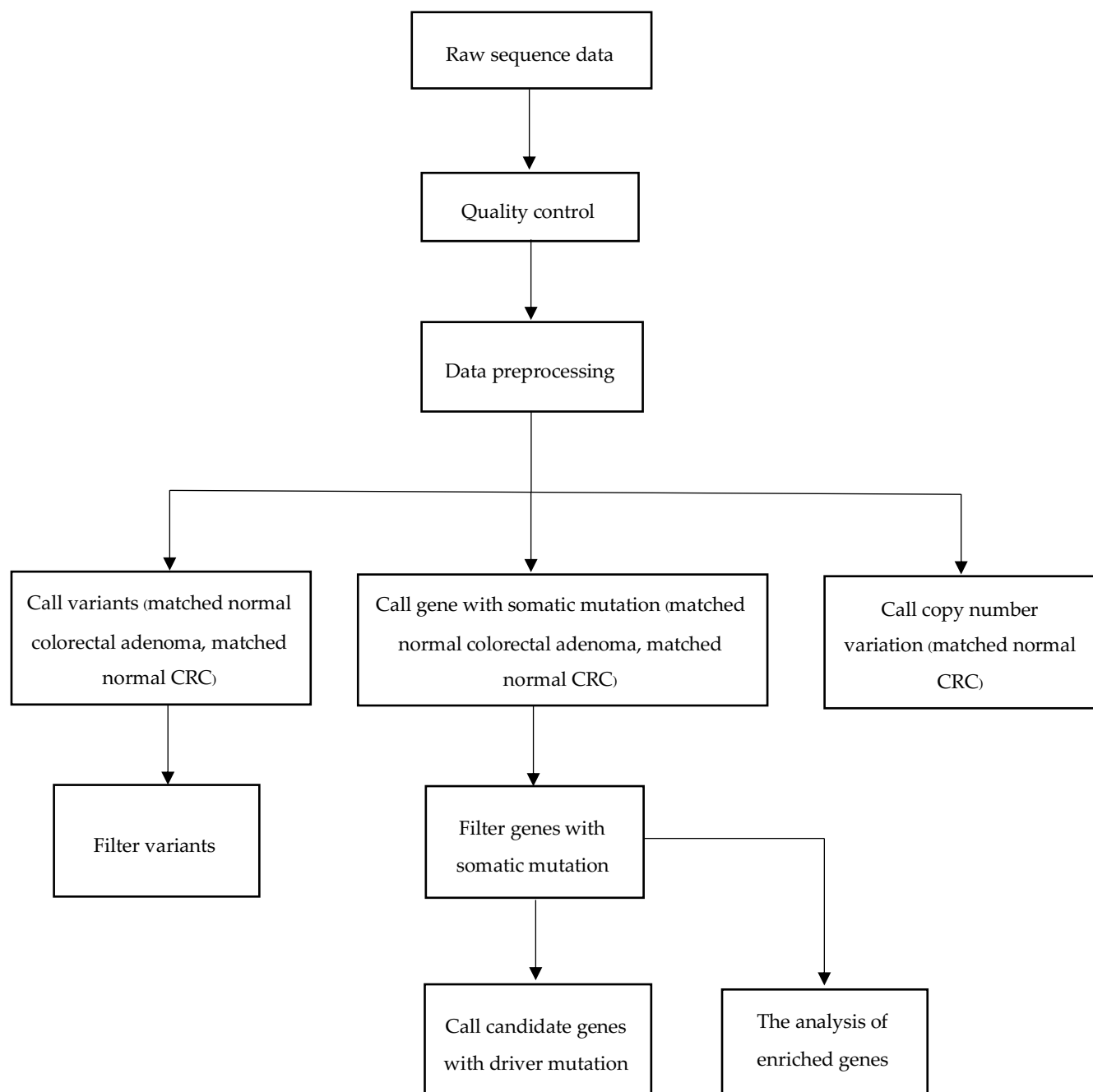

Figure S1: Data flow of the project

Supplement: Supplementary file 1 [file cancers-11-00977-s001.zip › supplementary_file/Supplementary Figure1.pdf]
